# Exploring the One Health–One Welfare nexus and zoonoses

**DOI:** 10.1016/j.soh.2025.100128

**Published:** 2025-10-13

**Authors:** Bernabé Vidal, Lorenzo Verger, Gustavo J. Nagy

**Affiliations:** a Programa de Posgrado en Ciencias Ambientales, Instituto de Ecología y Ciencias Ambientales (IECA), Facultad de Ciencias, Universidad de la República. Iguá 4225, Montevideo, Uruguay; b Unidad de Salud Pública Veterinaria, Facultad de Veterinaria, Universidad de la República, Ruta 8 (kilómetro 18) y Ruta 102, Montevideo, Uruguay; c Unidad de Zoonosis y Vectores, Ministerio de Salud Pública, Av. 18 de Julio 1892, Montevideo, Uruguay

**Keywords:** Multi-species governance, One Health, One Welfare, Public health, Uruguay, Zoonoses

## Abstract

**Background:**

One Welfare expands the One Health approach by integrating animal welfare, human wellbeing, and environmental sustainability into a single framework. Despite growing global recognition of One Welfare as a governance tool to address zoonotic risks, societal vulnerabilities, and ethical challenges, its practical implementation remains limited in most regions. Uruguay provides a relevant case to explore how systemic gaps in animal welfare regulation can undermine public health resilience and zoonotic disease control.

**Methods:**

We analyzed Uruguay's position in international animal welfare indexes, reviewed national animal welfare legislation and educational strategies, and conducted a systematic assessment of One Welfare-related conditions. Additionally, we identified structural risks and governance deficits linked to inadequate animal welfare practices, including zoonotic risks, through a targeted literature review and policy analysis.

**Results:**

Despite global advances in multi-species health governance, Uruguay shows legislative fragility and fragmented institutional frameworks. Key failures include outdated laws, inadequate animal-ethical perception, uncontrolled companion animal populations, deficient sterilization practices despite legal mandates, overwhelmed shelters, rising animal–vehicle collisions, culturally entrenched but underregulated hunting, illegal wildlife trade, limited veterinary oversight, and painful routine farm practices with minimal anesthesia. Gaps in surveillance and biosecurity amplify underreported zoonotic threats, reflecting a structural disconnect between One Welfare principles and policy implementation.

**Conclusion:**

To move from aspirational discourse to actionable strategies, One Welfare must be embedded as a governance instrument that enables multi-species stewardship and integrated health systems. Uruguay exemplifies the pressing need for comprehensive, intersectoral solutions to mitigate zoonotic risks, enhance public health, and align national policies with global sustainability agendas.

## 1 Introduction

The One Welfare framework emphasizes the interdependence of animal welfare, human well-being, and environmental health, promoting multi-sectoral collaboration that transcends disciplinary silos [1]. Despite growing recognition in international forums and pilot projects, One Welfare remains unintegrated in national research and policy agendas. Its global implementation requires science-based strategies that connect local realities with global goals, tackling shared issues like zoonoses, climate change, and welfare gaps. This calls for coordinated efforts among the World Organisation for Animal Health (WOAH, formerly OIE), the World Health Organization (WHO), and the Food and Agriculture Organization of the United Nations (FAO), supported by transdisciplinary research that links ecological, economic, and ethical domains [2].

One Health often overlooks the socioecological complexity and the well-being of non-human animals and their environments. At the same time, One Welfare, rooted in ethical understandings of welfare, offers a framework to broaden and guide One Health toward greater equity and inclusivity. Together, these approaches can extend understanding to encompass the entire biosphere, enabling new tools and ethical insights to promote holistic health and sustainability across all life forms [3].

Scientific evidence increasingly confirms animal sentience—including cognition, emotion, and communication—recognized by the European Union (EU) laws and the WOAH. Intensive farming often restricts natural behaviors and positive emotions essential to animal welfare. Recognizing sentience demands a shift toward regenerative, agroecological, or organic systems that reduce suffering, meet animals’ needs, and improve Planetary Health by enhancing soils, lowering impacts, and yielding more nutritious food [4].

Building on these imperatives, it is essential to note that globally, including in Uruguay, the relationship between One Welfare and zoonotic risks remains underexplored. This review examines animal welfare conditions, zoonotic disease threats, and institutional gaps, positioning One Welfare as a diagnostic and strategic axis to enhance interspecies health, policy coherence, and public health resilience in response to emerging challenges.

## 2 Methods

This review examines the One Health–One Welfare nexus in Uruguay, focusing on how deficits in animal welfare governance are linked to zoonotic risks and public health vulnerabilities. Uruguay is a small but highly productive livestock-based country with a centralized governance system, a strong state presence, and a relatively low population density. It was deliberately chosen as a case study because its scale and institutional configuration make it a revealing model for understanding structural challenges in animal welfare governance.

To contextualize the animal welfare situation in Uruguay, we reviewed its standing in international indexes (Voiceless Animal Cruelty Index [VACI], Animal Protection Index [API], and Global Animal Law [GAL]), its legislation, and institutional frameworks.

We conducted a systematic literature review in accordance with the Preferred Reporting Items for Systematic Reviews and Meta-Analyses (PRISMA) guidelines. We searched Google Scholar, SciELO, and PubMed, with a preference for Google Scholar due to its access to peer-reviewed and relevant grey literature. As an exploratory review without meta-analysis, it was not registered in the International Prospective Register of Systematic Reviews (PROSPERO) and did not apply formal bias tools. The search formula was: (“Bienestar animal” OR “Animal welfare”) AND (“Uruguay”). Although concise, this formula was applied across the entire text of indexed documents (not limited to title, abstract, or keywords), ensuring that any mention of animal welfare in relation to Uruguay was retrieved. The bilingual search (Spanish and English) further expanded the scope, capturing studies in both local and international contexts. In addition to peer-reviewed publications, grey literature, such as theses, technical reports, and institutional documents, was systematically included. While other terms (e.g., “animal protection” and “animal ethics”) occasionally appear in discourse, “animal welfare/*bienestar animal*” remains the most widely used descriptor in Uruguay and internationally, particularly following the global surge in usage of the term over the past decade. Therefore, the chosen terms ensured both sensitivity and specificity for the scope of this review.

The Google Scholar search yielded over 8000 results, with relevance declining after approximately the first 400 entries; we systematically screened the first 800 records. SciELO and PubMed returned fewer unique items (6 and 92, respectively), with most items overlapping with those found in Google Scholar. We included all platforms for transparency. While we did not use formal risk-of-bias tools, predefined inclusion and exclusion criteria (Table 1) ensured thematic and methodological coherence.

**Table 1:** Inclusion and exclusion criteria.

Inclusion	Exclusion
Documents published between January 2015 and July 2025 (articles, reports, reviews, analyses, surveys, and technical series)	Documents published before 2015
In all languages	–
Focused on Uruguay	Not focused on Uruguay
Focused directly on animal welfare or abuse	Not focused on animal welfare or abuse (or only mentioned as a superficial topic, with primary emphasis on productive parameters, proof-of-concept tools, or experimental trials unrelated to welfare outcomes)

Documents were included if published between January 2015 and July 2025, in any language, provided they focused on Uruguay and directly addressed animal welfare or abuse. Those published before 2015, not focused on Uruguay, or mentioning animal welfare only superficially (e.g., productive parameters or unrelated experimental trials) were excluded (Table 1). The regulatory context justified this cut-off: although Uruguay passed its general animal welfare law in 2009, the effective enforcement only began with its regulatory decree in 2014 (Decree 204/014). Therefore, 2015 was chosen as a turning point marking the start of a more consolidated institutional framework for animal welfare in Uruguay.

**Fig. 1:**
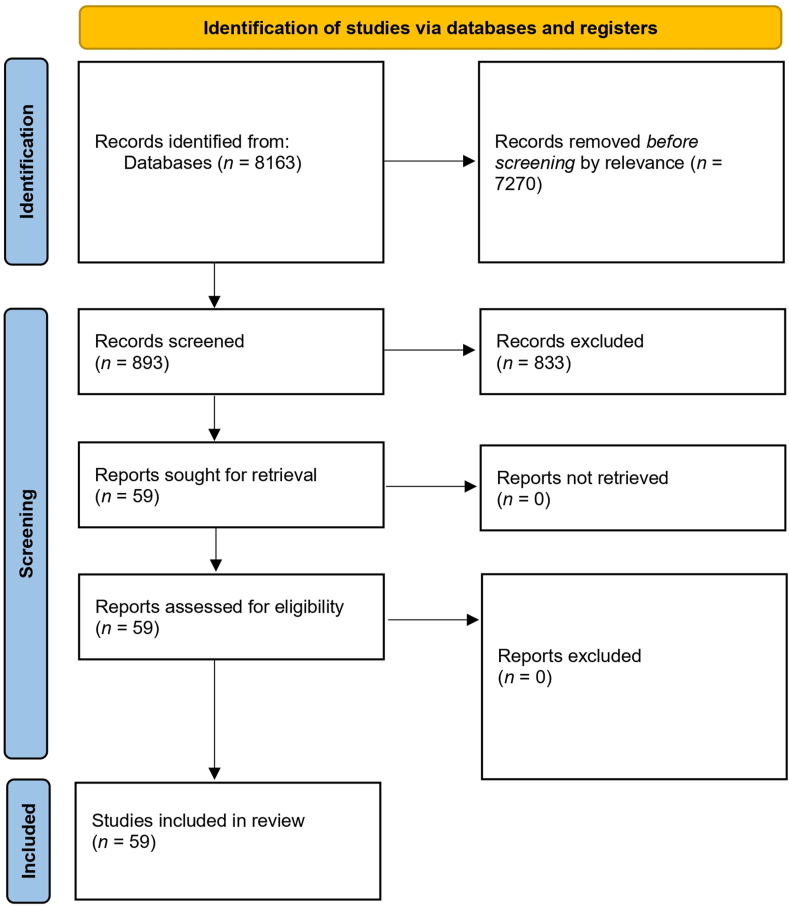
Flowchart of the literature search and selection process. A total of 8163 records were identified, of which 59 studies were finally included after screening and eligibility assessment. All 59 studies are available in the Supplementary Material. For clarity, the main text results section highlights the 34 most relevant publications that best illustrate the situation of One Welfare and animal welfare in Uruguay, while additional works are treated in the Discussion section.

A flowchart was created to outline the inclusion process and the subsequent classification (Fig. 1).

We also examined zoonoses linked to poor animal welfare—an often-overlooked intersection—underscoring the need to reframe animal-related issues within health and policy agendas. Finally, we assessed scientific and sociopolitical readiness for the broader adoption of One Welfare.

In addition to the systematic review, complementary contextual literature was integrated to broaden the scope beyond the animal welfare-centered search string. Most of the zoonosis content originates from these sources; similarly, additional literature on environmental issues, education, and ethics was incorporated to capture One Welfare–One Health–Planetary Health interactions that were not fully addressed by the systematic search. All complementary sources were screened for direct relevance to Uruguay and to multi-species welfare-health interfaces.

## 3 Results

**Fig. 2:**
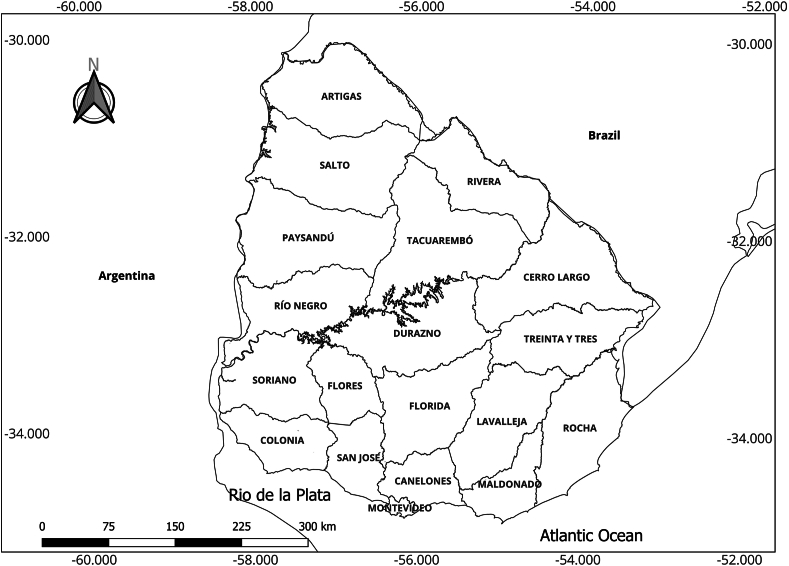
Uruguay map showing the political-administrative division (19 departments, source: the authors, Software QGIS v3.28.5, Data Source: INE, Uruguay. CRS: WGS 84/UTM Zone 21S, EPSG:32721).

In Uruguay, a country divided into 19 administrative departments, eco-social approaches were primarily applied (Fig. 2) [5].

### 3.1 Uruguay's current animal welfare landscape: international indexes and national framework

Uruguay scored a D in the 2020 API [6], below Mexico, India, and most European countries (high visible API benchmarks from different regions with recent legal reforms, as sentience recognition), mainly due to the lack of legal recognition of animal sentience and severe farm welfare gaps: no species-specific regulations, tolerance of painful procedures (e.g., piglet castration and beak trimming), no limits on stocking density or transport duration, and legal use of farrowing crates and unstunned halal slaughter. The API urges comprehensive legislative reform. Additional API recommendations include (1) banning cosmetic animal testing, (2) improving conditions for working and recreational animals, and (3) enhancing oversight for captive animals.

Although Uruguay has partially adopted WOAH standards, enforcement remains weak and inconsistent. In the VACI (2020) [7], Uruguay ranked 13th out of 50 in the “adequate” (C-tier) category, alongside Germany and Algeria—an improvement from 31st place in 2017. However, the VACI continues to highlight (out of 50 countries analyzed) (1) very high levels of animal consumption (36/50), (2) a strong economic dependency on animal production, (3) high animal protein intake in farm diets (30/50), (4) weak legislative protections for farmed animals, (5) and the highest number of farm animals per capita (50/50). The report concludes that greater dependency is correlated with increased animal suffering and structural gaps in aligning with international welfare standards.

Finally, the GAL index [8] classifies Uruguay as having a moderately developed legal framework, acknowledging the existence of formal laws but pointing to limited protections and ineffective enforcement. This supports the broader diagnosis of symbolic progress without systemic transformation. Uruguay's performance in international animal welfare rankings reveals persistent legal, structural, and ethical shortcomings. Table 2 summarizes Uruguay's relative position in South America, highlighting regional differences.

**Table 2:** Comparative performance of Uruguay and selected Latin American countries in three international animal welfare indexes: Animal Protection Index (API), Voiceless Animal Cruelty Index (VACI), and Global Animal Law (GAL).

Index	Grade/category							
	Uruguay	Argentina	Brazil	Chile	Peru	Colombia	Venezuela	Mexico
**API**								
**General**	D	E	D	D	D	D	E	C
Animal sentience is formally recognized	C	D	C	B	B	A	E	C
Laws against causing animal suffering	C	D	C	B	B	B	D	B
Protecting animals used in farming	F	F	D	F	G	E	G	D
Protecting animals in captivity	C	F	C	E	E	F	F	C
Protecting companion animals	C	D	C	D	D	D	D	B
Protecting animals used for draught and recreation	F	D	F	G	D	F	F	D
Protecting animals used in scientific research	C	E	C	C	C	C	D	C
Protecting the welfare of wild animals	E	E	B	D	F	E	C	C
Government accountability for animal welfare	B	D	D	D	C	E	E	C
WOAH (formerly OIE) animal welfare standards	D	D	D	E	E	D	G	E
**VACI**								
**General**	13 C	46 F	43 F	41 E	32 E	30 D	37 E	19 D
Producing cruelty	17	36	42	35	47	28	29	22
Consuming cruelty	22	48	47	38	10	34	21	32
Sanctioning cruelty	18	32	22	28	29	23	41	14
**GAL**^a^								
**World at national level (CASE)**	Case 2	Case 6	Case 6	Case 2	Case 2	Case 5	Case 2	Case 2

a Note: According to the GAL classification, **Case 2** refers to countries with a fundamental national law (e.g., anti-cruelty legislation or penal code provisions) together with new animal welfare legislation; **Case 5** refers to countries with a fundamental national law plus a national civil code provision granting animals a new legal status; and **Case 6** refers to countries with a fundamental national law and a provincial or local constitutional principle concerning animals. Abbreviations: WOAH, World Organisation of Animal Health; OIE, Office International des Epizooties.

Compared with neighboring countries, Uruguay presents a paradoxical profile. The API and GAL place it in a weak position (general grade: D; GAL = Case 2), with significant gaps in farming, captivity, and animal use for draught or recreation. Sentience is still not formally recognized, and the intermediate “C” grade reflects partial rather than explicit progress. In contrast, the VACI points to lower cruelty production and consumption (general grade:13 C), positioning Uruguay ahead of Argentina (46 F) and Brazil (43 F). This mismatch—moderate practical indicators co-existing with fragile and outdated legislation—suggests a delicate balance. At the regional scale, significant data gaps persist, with countries such as Ecuador, Bolivia, Paraguay, Suriname, Guyana, and several Caribbean nations lacking comparable metrics. What emerges here is not a conclusion but a warning: if even Uruguay, often perceived as stable and centralized, already displays such inconsistencies, the broader One Health–One Welfare landscape across Latin America—and, by extension, in international contexts—remains highly uncertain, calling for regionally integrated and globally coherent strategies.

Against this backdrop, the country nevertheless made gradual legal progress: the National System of Protected Areas (SNAP; *Sistema Nacional de Áreas Protegidas*) was created in the 2000s [9], Animal Protection, Welfare and Ownership Law, No. 18471 (*Ley de Protección, Bienestar y Tenencia de Animales, Ley N° 18471*) was passed in 2009 [10], National Honorary Commission for Responsible Ownership and Animal Welfare (CONAHOBA; *Comisión Nacional Honoraria de Bienestar Animal*) was established in 2014 [11], welfare meat certification was promoted [12], and bans were enacted on dog racing [13], cosmetic surgeries [14], and loud fireworks [15]. The establishment of the National Institute of Animal Welfare (INBA; *Instituto Nacional de Bienestar Animal*) in 2020 marked a key institutional milestone [16]. Non-governmental organizations (NGOs) contribute through sheltering, adoption, reproductive control, conservation, education, and advocacy [[17], [18], [19]], indicating early structural change with long-term potential.

Despite progress, Uruguay still lacks formal recognition of animal sentience [6,7], perpetuating the outdated notion that animals are mere “things.” Law No. 18471 has had a limited impact, with scarce resources [20] and regional criticism compared to Mexico, Chile, and Colombia [21]. Animals remain “objects of rights” and private property under Decree 204/2017. Sanctions for abuse—beating, poisoning, killing—are primarily administrative (e.g., fines and license suspensions), with limited deterrence. Criminal protection is urgently needed, and legal reforms are under discussion [22,23].

Uruguay faces a critical overpopulation of companion animals—estimated at around 300,000 stray dogs and cats (for a population of 3.5 million humans), roughly triple the WHO threshold—driven by gaps in policy, education, and cultural empathy. While official estimates remain limited, the 2023 national census reported that 36.5 % of households own dogs and 8 % own cats [24]. A 2017 survey by the National Honorary Commission for Responsible Ownership and Animal Welfare (COTRYBA; *Comisión Nacional Honoraria de Tenencia Responsable y Bienestar Animal*) highlighted systemic shortcomings [25]. Overwhelmed shelters and widespread free-roaming persist, with additional issues such as socially gender-biased sterilization practices (female-focused), poor feeding, and inadequate carcass disposal [26].

Key institutions, such as the National Veterinary Academy, the Faculty of Veterinary Medicine, and the Veterinary Medicine Society (SMVU; *Sociedad de Medicina Veterinaria del Uruguay*), link this to zoonoses, accidents, aggression, and rural abandonment, urging cultural change, unified policies, rabies vaccination, improved leptospirosis coverage, a national leishmaniasis plan, and systematic deworming [27,28].

Veterinary and agricultural actors strongly criticize INBA's performance. The SMVU denounced its lack of transparency, ineffective results, and marginalization of veterinary expertise [29]. Likewise, rural entities (i.e., Rural Association of Uruguay [ARU; *Asociación Rural del Uruguay*] and Rural Federation of Uruguay [FRU; *Federación Rural del Uruguay*]) expressed concern over insufficient action regarding predation and highlighted the viability crisis of the ovine sector [30,31].

Wildlife trafficking reflects growing demand, legal gaps, and weak controls, with exotic companion animals (e.g., hedgehogs, snakes, and tropical birds) and native species heavily targeted. From 2017 to 2019, 2500 animals across 109 species were seized—81 % native, 67 % non-CITES (species or products not listed under the *Convention on International Trade in Endangered Species of Wild Fauna and Flora* [CITES], and therefore not directly regulated by it) [32]. National Directorate of Biodiversity and Ecosystem Services (DINABISE; *Dirección Nacional de Biodiversidad y Servicios Ecosistémicos*) is the only agency with a dedicated unit; customs, police, and navy lack training, infrastructure, and coordination. There is no unified data system or rehabilitation center [32]. NGOs advocate for bans on wild companion animals, while breeders propose regulated captive breeding [32]. Rescues are recurrent and often involve the care of hundreds of animals [33,34].

In light of these findings, animal welfare appears to be undermined not only by legislative or institutional weaknesses but also by deeper gaps in educational and cultural perceptions.

### 3.2 Zoonotic risk pathways in Uruguay (2015–2025)

#### 3.2.1 Topic summary of published documents since 2015 in Uruguay

**Fig. 3:**
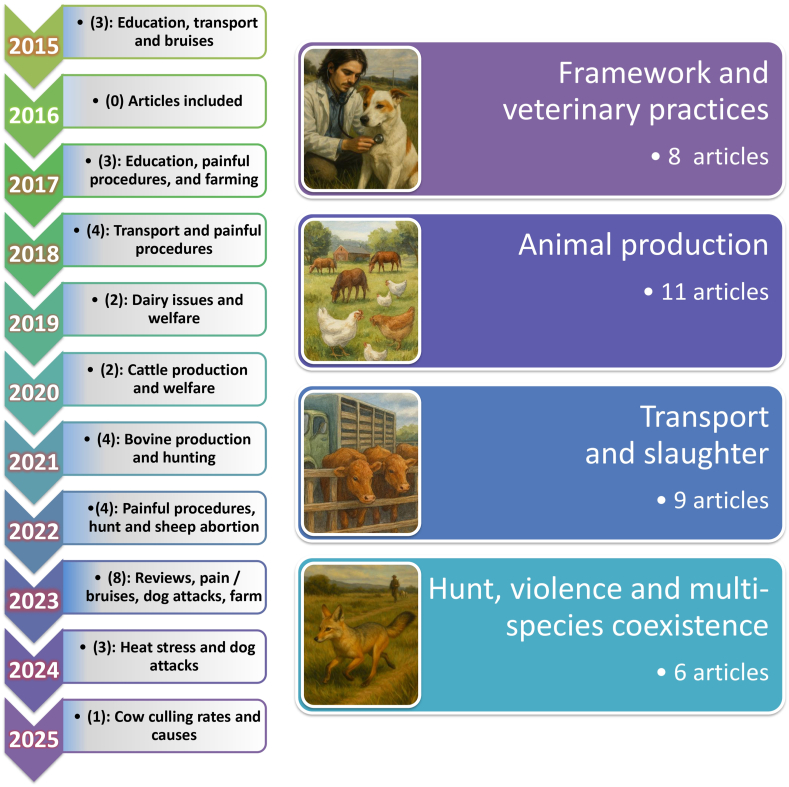
Animal welfare and One Welfare-related topics in Uruguay from 2015 to 2025.

This section summarizes the most influential publications on animal welfare and One Welfare-related topics in Uruguay since 2015 (34 articles, Fig. 3), providing context for issues addressed in subsequent sections.

##### 3.2.1.1 Framework and veterinary practices

Comparative legal studies confirm that current welfare laws in Uruguay remain weak, with barriers to enforcing animal legal personhood primarily due to scarce resources [20]. Broader reviews have linked animal welfare to One Health and One Welfare, distinguishing it from animal rights while examining consumer influence, welfare indicators, institutional milestones, and common failings such as transport bruising [35]. These structural deficits are mirrored in the professional sphere: veterinarians working in cattle and meat production widely acknowledged the need for greater animal welfare training. They strongly supported its inclusion in curricula [36,37].

Despite these concerns, Uruguay has produced little academic scholarship on animal welfare. Research on ruminants began late and remains limited; until 2018, the country contributed only 0.35 % of global bovine and 0.58 % of ovine publications, a gap of nearly two decades compared with other regions, weakening Uruguay's international standing [38]. Veterinary practices illustrate similar shortcomings: disbudding and dehorning are widespread, with most procedures performed without analgesia due to time and cost constraints. Few practitioners recognized the importance of pain relief, exposing gaps in ethics, regulation, and training [39,40]. Companion animal medicine shows a comparable pattern, with perioperative pain routinely undertreated. Many veterinarians rely solely on mild opioids or nonsteroidal anti-inflammatory drugs (NSAIDs), constrained by cost, regulation, and insufficient education, revealing a persistent mismatch between the severity of pain and the adequacy of treatment [41].

##### 3.2.1.2 Animal production

The production sector reflects the same systemic weaknesses. Dairy research has shown that cows with access to pasture display more natural behaviors and improved comfort compared to stall-kept animals, although further investigation is needed [42]. Hoof disease trends further illustrate shifting challenges: while most cases between 2001 and 2004 were infectious, by 2016, injuries and diet had become predominant, with white line disease alone accounting for 67.61 %. Smaller herds were most severely affected, emphasizing the need for regular inspection [43]. By 2015, hoof conditions—including heel damage, overgrown toes, white line splits, and swelling—affected 4.93 % of animals, with variations in prevalence by age and lactation stage [44]. Suggested responses included improved facilities, routine trimming, foot baths, and closer attention to younger cows.

These findings align with broader dairy farm assessments, which have revealed persistent welfare failures, including lameness, poor pathways, inadequate shade and water, high culling and disease rates, and elevated calf mortality before weaning [[45], [46], [47], [48]]. Such problems are rooted in poor colostrum management, inadequate hygiene, and insufficient veterinary support. Feedlot research confirms the same vulnerabilities under intensive systems. Shade improved behavior, rumen pH, feed efficiency, daily gain (+13 %), and carcass weight, while its absence during heat extremes led to mortality [49]. These outcomes underscore the crucial role of shade, water, dietary adjustments, and specialized management. Trials in Salto using the Heat Load Index showed shade improved weight gain under moderate conditions. However, they were insufficient during severe heatwaves, demonstrating that natural shade remains indispensable in rangeland systems [50].

Reproductive health adds further complexity. Necropsy of ovine abortion cases (2015–2021) documented *Toxoplasma gondii*, *Campylobacter fetus*, and dystocia as leading causes, with fetal suffering frequently evident. Many of these cases were preventable, exposing serious welfare and zoonotic risks while also highlighting the value of abortion surveillance for both reproduction and public health [51]. Bovine viral diarrhea virus (BVDV) was also identified, while other causes remained undetermined or non-infectious. Gaps in vaccine access and diagnostics compounded the risks, underlining the need for stronger reproductive health programs [52].

##### 3.2.1.3 Transport and slaughter

The end of the production chain introduces further vulnerabilities. Studies of cattle transport to abattoirs have revealed frequent bruising, even when drivers have received training and prods are in use [[53], [54], [55], [56], [57]]. Recommendations focused on upgrading vehicles, improving handling, and expanding training, with innovations such as PROGAT® (a protection system for livestock during transport) and flag use helping to reduce injuries and carcass losses [54]. Still, high rates of bruising persisted, especially in older females, with significant consequences for both welfare and meat quality [[53], [54], [55], [56], [57]].

Later investigations confirmed that, while trucks often met technical standards, welfare issues remained entrenched. Stress, bruising, excessive use of prods, and poor handling were recurring problems that remained unaffected by existing driver training. This highlights the need for stricter audits, revised handling protocols, and infrastructural improvements [58,59]. Diagnostic challenges added to the difficulty: most tools could not accurately age bruises, but infrared thermography offered promise by identifying injuries older than 12 h, providing new options for monitoring and traceability [60]. Research on pasture-finished steers complemented this picture, showing that transport caused physical stress, while lairage periods of 15 h improved carcass pH and recovery. Calmer animals adapted better, reinforcing the importance of temperament and lairage management for both welfare and productivity [61].

##### 3.2.1.4 Hunt, violence, and multi-species co-existence

Outside production, societal conflicts further expose weak governance. Attacks by free-ranging dogs remain widespread, reflecting gaps between the Rural Code, which authorizes killing to protect livestock, and the animal welfare law, which prohibits cruelty [62]. Nearly half of surveyed farmers (49 %) reported losses and stress, underscoring the scale of the problem [63]. These conflicts, rooted in weak governance and education, have triggered calls for legislation, cultural reform, and interdisciplinary responses [64].

Official data from 2018 to 2019 documented 848 incidents, with 7163 sheep injured or killed by stray dogs. These figures suggest underreporting, geographical unevenness, and institutional shortcomings, reinforcing the need for evidence-based responses [65]. Tensions extend into hunting, where disputes between hunters and activists reveal cultural divides. Hunters regard dogs as partners, while activists view the practice as cruel. Legal ambiguities leave hunting dogs largely unregulated, while wild boars are classified as pests. Fragmented institutions prevent coordination between welfare, conservation, and governance [66].

Debates over wild boar hunting illustrate the same gaps: ambiguous ethics, policy voids, and disjointed enforcement mechanisms. Together, these examples reveal how multi-species conflict amplifies the weaknesses already visible in production systems [67].

##### 3.2.1.5 Integrative synthesis

Taken together, these findings reveal consistent and systemic weaknesses. Across veterinary practice, production, slaughter, and multi-species co-existence, Uruguay shows recurrent patterns of inadequate pain control, poor handling, high disease incidence, and weak regulatory enforcement. These failures undermine both welfare and productivity, leaving animals immunologically stressed, susceptible to chronic pain, wounds, infections, and parasites, all within a system of fragile veterinary oversight.

Beyond technical deficits, ethical neglect and educational gaps perpetuate cycles of intra- and inter-species violence, reinforcing harm and weakening resilience. Research remains heavily biased towards productive species, overlooking the interconnected welfare of companion animals, wildlife, strays, and humans.

The result is a porous web of vulnerabilities, where livestock, domestic animals, wildlife, humans, and vectors interact without adequate safeguards in place. This creates fertile conditions for the emergence and persistence of zoonotic diseases, especially under outdated co-existence norms and inadequate surveillance. The following section examines emblematic diseases within this fragmented One Welfare–One Health landscape, showing how governance failures, welfare deficits, systemic violence, and biased research intersect to sustain multi-species risks across animals, people, and ecosystems.

#### 3.2.2 Key examples of zoonoses linked to the One Welfare situation in Uruguay

##### 3.2.2.1 Livestock**–**wildlife bacteria

Livestock–wildlife bacteria such as *Brucella* spp. and *Mycobacterium* spp. persist across livestock, wildlife, and humans, underscoring the deeply interconnected nature of One Welfare concerns. Human brucellosis still occurs sporadically in Uruguay [68]. At the same time, the first pulmonary *Mycobacterium bovis* cases were identified in 2012, linked to direct contact with zoo animals, handling of infected meat, and the consumption of raw milk [69]. Wild boar hunting, frequently conducted with dogs and largely lacking adequate legal oversight, has been directly associated with the transmission of brucellosis, tuberculosis (TB), and leptospirosis [66,67,70].

The consequences extend beyond simply hunting or farming. TB outbreaks have caused significant mortality among wild animals in reserves [71]. Dairy farm studies indicate that only 38.7 % of farms receive regular veterinary care [48], resulting in persistent gaps in prevention and response. Deficiencies in zoonotic reporting and insufficient training for farm workers further compromise control [72,73]. Together, these findings reveal how fragile oversight at the human–animal–environment interface facilitates the persistence of bacterial diseases across species.

##### 3.2.2.2 Parasites

Parasitic infections remain widespread in livestock, companion animals, and wildlife, with impacts documented across diverse contexts [[74], [75], [76], [77], [78], [79], [80]]. *Cryptosporidium parvum* is particularly concerning, as it commonly infects dairy calves [77] and contaminates water sources used for human consumption, exemplifying the One Welfare link between animal, human, and environmental health. Surveillance of wild and feral boars revealed *Toxoplasma gondii* in 53.9 % of individuals sampled across 11 of 13 departments [78]. In 2021, screwworm infestation was confirmed for the first time in a feral boar in Uruguay [76], further illustrating the porosity of boundaries between domestic and wild populations.

Parasitism is especially prevalent in marginalized regions [74], where inadequate veterinary services, outdated regulations, poor staff training, and weak monitoring systems exacerbate risks [6,48,72,73]. In response, veterinarians frequently advocate for mass deworming across species [26]. Collectively, these findings highlight how parasitic threats exploit structural weaknesses, with direct consequences for animal productivity, human health, and ecological resilience.

##### 3.2.2.3 Avian/equine zoonoses

Avian and equine diseases illustrate how welfare failures and ecological dynamics converge to shape zoonotic risk. In poultry, the absence of regulation on battery cages [6,7], combined with persistent welfare gaps [40], increases the likelihood of *Salmonella* spp. infection [81], while illegal bird trafficking continues to expand [32], multiplying welfare harms and zoonotic exposure.

Western equine encephalitis (WEE) virus, a mosquito-borne virus with birds as the primary reservoir, illustrates the fragile balance between welfare, environment, and health. Transmission occurs when mosquitoes bite horses and humans, and shifts in bird or mosquito populations amplify cross-species risks [72]. However, these epidemiological dynamics unfold within an equine sector already marked by structural neglect. Deficiencies in slaughter and transport [82,83], as well as high-risk competitive events [[84], [85], [86]], expose horses to cumulative stress and injury. At the same time, widespread abandonment and the proliferation of free-roaming horses [87,88] further compromise both welfare standards and disease control. This convergence highlights how zoonotic threats, such as WEE, are inextricably linked to the broader neglect of equine populations, underscoring the interdependence between One Welfare and One Health.

Taken together, these factors demonstrate that zoonotic threats, such as WEE, do not emerge in isolation, but within a broader framework of deficient equine welfare, environmental change, and porous human–animal interfaces—capturing the essence of the One Welfare–One Health nexus.

##### 3.2.2.4 Canine-feline-wildlife zoonoses (hunt and co-existence)

Zoonoses involving companion animals and wildlife highlight further vulnerabilities. Rabies, leishmaniasis, and sporotrichosis continue to circulate, with the latter now recognized as a fungal infection affecting cats, dogs, and wild animals like armadillos [[89], [90], [91]]. Urban bats, stray dogs, and cats pose a risk to rabies transmission [92] in a context where Uruguay is estimated to host approximately 300,000 stray animals nationwide [26]. Preventive measures remain limited: rabies vaccination is rare, and there are no integrated programs for leishmaniasis, weakening disease control. The condition spreads most readily in areas with poor infrastructure and untreated animal reservoirs [28,93,94].

Emerging diseases illustrate the same patterns. Most human sporotrichosis cases in Uruguay are associated with hunting armadillos [91], and cat bites pose an additional public health concern [95]. Rising wildlife trafficking [32] also increases the likelihood of disease spillover. Together, these examples demonstrate how poverty, urbanization, and close human–animal interactions create a fertile ground for zoonotic persistence.

##### 3.2.2.5 Integrative synthesis

Across bacterial, parasitic, avian, equine, and companion–wildlife zoonoses, a consistent pattern emerges: inadequate veterinary coverage, outdated regulations, poor surveillance, weak reporting systems, and insufficient training converge with systemic welfare deficits, wildlife trafficking, and risky human–animal practices. These One Welfare shortcomings directly shape the persistence and spread of zoonotic diseases in Uruguay.

**Fig. 4:**
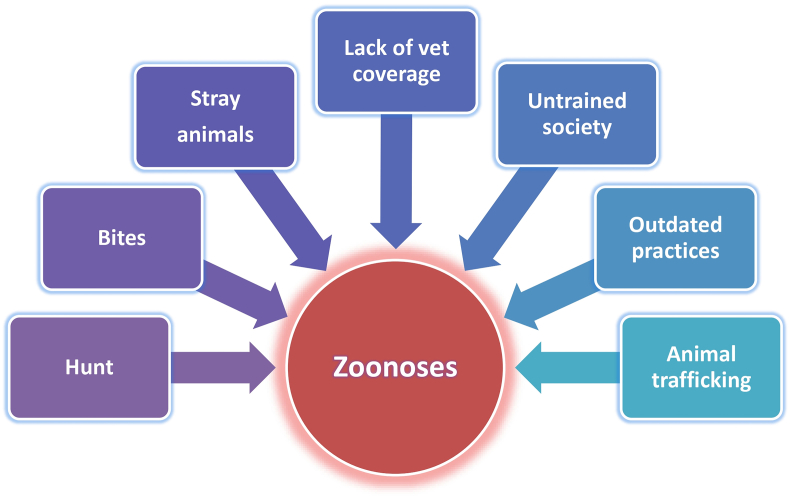
Direct One Welfare factors influencing zoonoses. Abbreviation: vet, veterinary.

Fig. 4 summarizes the main One Welfare factors influencing One Health in this context, highlighting how systemic weaknesses link animal suffering with public health risks and ecological vulnerability.

#### 3.2.3 Environment issues (Planetary Health–One Welfare–One Health interface)

Although institutional and academic debates in Uruguay rarely integrate environmental pressures into the animal welfare agenda, these dimensions are inseparably linked. Pasture degradation, climatic extremes, water crises and land-use change all affect productivity, welfare, and disease dynamics. Framing these issues through the lens of Planetary Health reveals how ecological stressors and multi-species suffering converge, demanding a broader One Welfare–One Health perspective that extends beyond traditional legal and veterinary boundaries.

Pasture condition and climate pressures shape both productivity and welfare. Overgrazing, poor stocking management, and climatic stress degrade sward health, reproductive outcomes, and animal condition, despite the availability of national tools such as the CONEAT (*Comisión Nacional de Estudio Agronómico de la Tierra*) soil index. Ecological overcapacity continues to erode pasture quality and herd performance [[96], [97], [98], [99]]. Stagnant pregnancy (73 %) and weaning (62 %) rates reflect the combined effects of pathogens (*Campylobacter fetus*, *Leptospira* spp., BVDV), environmental stress, and poor body condition. Static pregnancy checks mask seasonal losses, underscoring the need for dynamic, welfare-integrated monitoring [100].

Aquatic risks mirror terrestrial degradation. Cyanobacterial blooms—driven by eutrophication, climate change, and inadequate watershed management—pose escalating threats. Toxins (e.g., microcystins and saxitoxins) have been detected in cattle and are associated with fish die-offs [[101], [102], [103]]. Institutional responses frequently overlook animal impacts due to the absence of species-specific protocols, insufficient riparian buffers and limited access to safe water [102].

Heat and water stress further expose the vulnerabilities of multi-species communities. Documented heatwaves have caused animal deaths in the absence of mitigation, while water crises and “summer-strike” mortality events highlight management gaps [49,50,[104], [105], [106], [107]]. Climate-adaptive, welfare-centered strategies—such as shade, abundant water, and low-stress handling—are therefore essential [49,84,108]. Silvopastoral systems can provide thermal relief and ecological co-benefits [[108], [109], [110]], although national concerns persist regarding toxic fungi in eucalyptus plantations responsible for emerging painful intoxications in livestock [111]. Risks extend beyond ruminants: horses are vulnerable during hot transport and competitive events, notably when lacking adequate rest, conditioning, and infrastructure [83,84]. Farms without adequate shade or trained staff face compounded impacts [73].

**Fig. 5:**
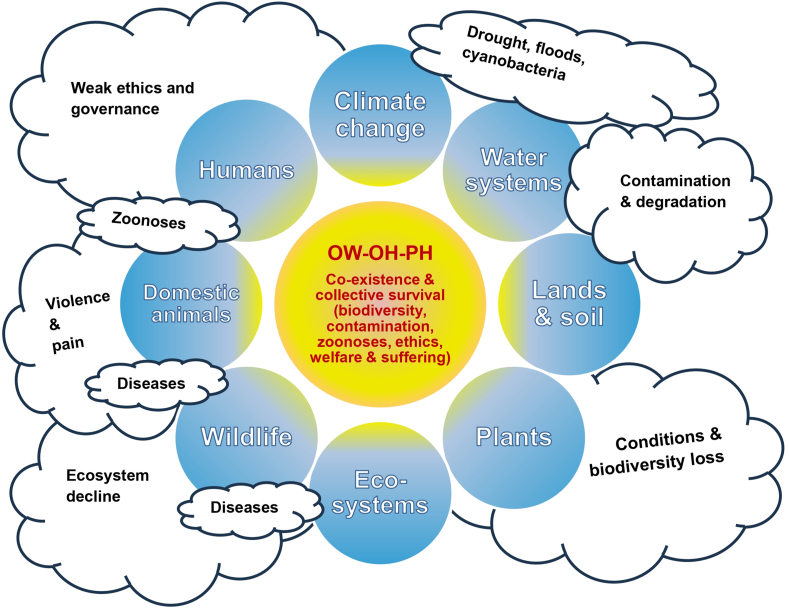
“Sun model” of the One Welfare–One Health–Planetary Health nexus in Uruguay. The central sun symbolizes the integrated One Welfare–One Health–Planetary Health nexus, representing co-existence and collective survival. Surrounding spheres highlight domains of interaction, while floating “clouds” mark threats—such as heat, drought, biodiversity loss, stray animals, weak governance, and zoonoses—that obscure the system. The figure shows how such pressures overshadow welfare and health, underscoring the need for integrated responses. Abbreviations: OW, One Welfare; OH, One Health; PH, Planetary Health.

Landscape and interface dynamics compound zoonotic risks. Hunting with dogs and wild boar management sustain porous interfaces for pathogen spillover [66,67,70]. In contrast, the overpopulation of some 300,000 free-roaming dogs and cats in a country of 3.5 million intensifies predation on native fauna and disease transmission, with a visible impact on the ovine sector [24,65]. Discarded abortuses, carcasses, and canine feces further contaminate shared environments [51,52,[74], [75], [76], [77], [78], [79], [80]], and parasitic threats, such as *Cryptosporidium parvum*, infect calves and pollute water sources [77]. Land-use change, afforestation and habitat fragmentation alter reservoir and vector dynamics; bats remain central for rabies at human–animal boundaries, now linked to expanding silviculture [92,112]. Wildlife trafficking magnifies these vulnerabilities under fragile enforcement and absent rehabilitation systems [[32], [33], [34]]. Taken together, these intertwined pressures confirm that animal welfare, ecosystem integrity and human health cannot be addressed in isolation. Their convergence within a One Welfare–One Health–Planetary Health frame illustrates how systemic vulnerabilities emerge when governance, environment, and husbandry fail to integrate. This complexity is summarized in Fig. 5, where the “sun model” depicts the central nexus of co-existence and survival, surrounded by key domains and overshadowed by environmental and institutional threats.

## 4 Discussion

This study highlights how fragmented awareness and institutional fragility continue to obstruct the effective implementation of One Welfare in Uruguay. International benchmarks confirm persistent weaknesses, including the lack of legal recognition of sentience, inadequate enforcement, and outdated confinement and transportation regulations [[6], [7], [8]]. However, paradoxes emerge: although Uruguay scores poorly in API (D) and GAL (2), its VACI grade (13 C) is more favorable than those of Argentina (46 F) and Brazil (43 F), suggesting a comparatively lower cruelty footprint. This inconsistency reveals not just uneven progress but a deeper structural misalignment between formal frameworks and practical outcomes. Suppose a country often regarded as centralized and relatively stable exhibits such contradictions. In that case, it is plausible that neighboring states—Ecuador, Bolivia, Paraguay, Suriname and several Caribbean nations—where comparable data are lacking, may face even greater vulnerabilities. These contrasts underscore the need for coordinated regional and international strategies, not only to close regulatory gaps but also to critically examine how indicators accurately capture (or fail to capture) the lived realities of welfare.

These gaps are not isolated but reveal structural inertia across scientific, veterinary, rural, and civic domains [[20], [21], [22], [23], [24], [25], [26], [27], [28], [29], [30], [31], [32],^54^,^62^]. The persistence of practices such as extracting pregnant mare serum—despite more than 60 synthetic alternatives [113,114]—shows how cruelty is normalized under weak regulation. Education reflects the same delay at multiple levels. In primary curricula, One Welfare and related frameworks remain largely absent [115], whereas in professional training, most veterinarians endorse animal welfare teaching yet continue to report limited preparation [36,37]. The divide is sharper in rural contexts, where a 2025 survey in Cerro Largo confirmed that producers receive some training but workers remain excluded, mainly, with only one in five exposed to welfare education [73]. Deficits also cut across species and sectors: companion-animal care still leans on euthanasia and psychotropics [116], livestock systems lack diagnostic rigor that could prevent fetal suffering [51], and stray-dog governance perpetuates cycles of overpopulation, violence, and zoonotic risk [64]. Taken together, these patterns signal not just technical shortcomings but an entrenched cultural tolerance for suffering that undermines both animal welfare and public health.

Scientific underproduction further entrenches these barriers. Until 2018, Uruguay contributed less than 1 % of global animal welfare publications in cattle, sheep, and goats [38], a delay of almost two decades compared with global trends. Although research output has since grown [114], much of it remains tied to productivity, leaving welfare as a secondary concern. International frameworks, such as those from the EU and WOAH, are formally adopted [45,48,117,118]; yet, without local adaptation, they function more as symbolic alignments than operational tools. The absence of species-specific protocols limits the generation of context-relevant evidence, constraining both policy design and enforcement.

These systemic weaknesses are reflected directly in production systems. In dairy, veterinary oversight remains inconsistent—by 2020, fewer than 40 % of farms had regular visits—while calf mortality, lameness, acidosis, and hygiene problems persisted [35,36,40,[43], [44], [45], [46], [47], [48]]. Pain management is routinely neglected: over 90 % of veterinarians perform procedures such as disbudding or dehorning without analgesia, often justifying the practice by citing costs or downplaying suffering, with tasks at times delegated to untrained staff. Similar patterns extend to beef cattle, where late castration remains widespread, and to companion animals, where many veterinarians report inadequate training and rely primarily on NSAIDs or mild opioids [6,7,[39], [40], [41],^73^]. Collectively, these practices reveal not only technical gaps but also a cultural minimization of animal pain that reinforces weak enforcement and slows the integration of One Welfare principles.

Transport and slaughter display the same entrenched neglect. Between 40 % and 70 % of carcasses show bruising—sometimes exceeding 90 %—with losses of nearly 1 kg per animal and most condemnations linked to pre-slaughter problems [[53], [54], [55], [56], [57], [58], [59],^82^,^83^]. Audits as early as 2012 had already documented poor stunning, drainage failures, and pervasive trauma, and more recent data confirm that, despite partial improvements, hematoma and abscess rates remain high, with 95 % of losses still traced to pre-slaughter conditions, also evident in ovine chains. Although initiatives such as PROGAT demonstrate promising tools for monitoring and reduction [54,[119], [120], [121]], their limited uptake highlights how systemic inertia continues to undermine both economic efficiency and ethical responsibility.

Wildlife and captive species expose the same structural deficits. In 2025, nearly four out of five welfare indicators for Geoffroy's cat remained unmet [122], underscoring the absence of systematic monitoring and enforcement. Chronic failures persist in farms and reserves [123,124], exemplified by the 2016–2017 *M. bovis* outbreak at the “Dr Rodolfo Tálice” Reserve, where more than 270 animals died amid delayed alerts, jurisdictional voids, and poor inter-institutional coordination [71]. Similar gaps also undermine companion-animal governance [[26], [27], [28]] and wildlife trafficking control [[32], [33], [34]], where enforcement capacity lags behind the escalating trade and recurring rescues. Together, these cases reveal not isolated oversights but a governance vacuum that compromises both conservation and public health within the One Welfare–One Health framework.

Cultural and social practices reinforce these systemic vulnerabilities. National meat consumption continues to exceed WHO recommendations, driven more by tradition and price than by ethical or environmental concerns [125,126]. Practices that cause intense suffering are often reframed in narrow economic terms. Cattle rustling, for example, remains primarily treated as a financial crime, despite the brutality it entails, with official reports declining from 1196 in 2020 to 369 in 2024. On-farm slaughter is similarly normalized, even as it raises concerns about both food safety and humane standards [127,128]. Harmful reproductive and handling methods, including early weaning and electroejaculation, persist despite documented physiological harm [129,130]. These examples illustrate how cultural inertia and economic priorities perpetuate a tolerance for cruelty, entrenching structural deficits and hindering the shift toward welfare-conscious systems.

Equine and canine welfare illustrate how institutional contradictions sustain neglect. In horses, beyond the extraction of pregnant mare serum [113], deficiencies in slaughter and transport persist [82,83], while traditional *jineteadas* (traditional rodeo-style horse riding competition), endurance events, and thoroughbred racing maintain high injury and mortality rates linked to poor rest and conditioning [[84], [85], [86]]. Dogs face equally fragmented governance: they are valued as companions yet rapidly reclassified as threats after livestock predation, under contradictory laws that simultaneously permit protection and lethal control [[64], [65], [66],^131^]. Structural gaps compound these tensions—high-risk breeds are frequently neglected [116], aggression cases resolved through obedience training, psychotropics, or euthanasia with little diagnostic support [116,132], and overpopulation perpetuated by abandonment and inadequate sterilization [[26], [27], [28]]. These patterns reveal how species central to cultural identity are managed through inconsistent norms that normalize suffering, fuel zoonotic risks, and erode the credibility of welfare policies.

Endemic zoonoses deepen these structural weaknesses. *Brucella* spp. and *Mycobacterium* spp. persist across species [69,70,72], with pulmonary *M. bovis* cases linked to raw milk, slaughter, and exposure in zoos since 2012 [69], and wildlife outbreaks causing mass die-offs [71]. Control remains inconsistent, undermined by outdated protocols, weak reporting, and aggravating factors such as the presence of stray dogs and wild boar hunting [[6], [7], [8],^26^,^28^,^62^,^65^,^66^,^70^]. Sheep abortions from *T. gondii*, *Campylobacter* spp. and BVDV further expose preventable fetal suffering [51,52]. Veterinarians themselves highlight systemic flaws: obsolete records, missing TB diagnosis protocols, poor training, antimicrobial misuse, and delays in culling over 1500 TB-positive cattle due to compensation barriers. In 2024, professional bodies, including SMVU and the Paysandú Vet-Centre, openly criticized the brucellosis criteria of the Ministry of Livestock, Agriculture, and Fisheries (MGAP), demanding multi-sectoral standards [71,133]. Their proposals—ranging from rabies vaccination in dogs to national leishmaniasis and deworming programs [28]—reflect both the urgency of reform and the chronic inertia of official responses.

Surveillance deficits stretch across parasites, production animals, and vector-borne diseases. In livestock and wildlife, unmanaged risks range from *Cryptosporidium* in calves to screw worms and *Toxoplasma* in feral pigs, compounded by unregulated hunting and over 100,000 annual animal–vehicle collisions that already account for nearly 10 % of road accidents [66,[76], [77], [78],^134^]. The poultry and equine sectors mirror these gaps, with welfare deficiencies in industrial flocks [6,7,53,81] and among free-roaming horses [87,88], both of which contribute to zoonotic vulnerability. Re-emerging infections make the consequences explicit: rabies has resurfaced, leishmaniasis continues to rise, and human sporotrichosis—linked to the illegal hunting of protected armadillos—underscores the socio-environmental failures that persist [72,91]. Together, these cases reveal how fragmented surveillance erodes both animal welfare and public health, preventing a coherent One Welfare–One Health response.

Hunting epitomizes the governance vacuum. Legal ambiguities blur the line between cultural tradition, pest control, and trafficking, creating a permissive space where cruelty and zoonotic risk are normalized. Wild boars, still legally classified as pests, are pursued with dogs that endure high injury rates and frequent abandonment, yet remain outside the scope of animal welfare protections [24,25,32,66,67,116,131]. This disconnection highlights how fragmented policy tolerates suffering while failing to contain ecological and health risks. Embedding a One Welfare perspective is crucial for reconciling ethics, public health, and sustainability within national governance.

Environmental pressures remain largely absent from Uruguay's welfare agenda, yet they are inextricably linked to animal and human health. Pasture degradation, overgrazing, and poor stocking management erode body condition and reproductive outcomes, with stagnant pregnancy and weaning rates reflecting the combined impact of pathogens, stress, and nutrition [[96], [97], [98], [99], [100]]. Water crises and cyanobacterial blooms—driven by eutrophication, weak watershed governance, and climate change—have generated toxins in cattle and fish; however, institutional responses rarely account for the impacts on animals [[101], [102], [103]]. Climatic extremes further expose gaps: heatwaves, “summer-strike” mortality, and inadequate access to shade, water, and low-stress handling reveal how adaptation remains reactive rather than systemic [49,50,84,[104], [105], [106], [107], [108]]. While silvopastoral systems offer welfare and ecological co-benefits [[108], [109], [110]], concerns such as eucalyptus-associated fungal intoxications persist [111], showing that integration requires more than technical fixes. Even equines, whether in transport or competition, illustrate how environmental stress compounds weak infrastructure and poor training [83,84]. These dynamics confirm that welfare, ecosystem integrity, and public health cannot be addressed in isolation; their convergence within a One Welfare–One Health–Planetary Health frame underscores how systemic vulnerability emerges when governance, environment, and husbandry remain fragmented.

The convergence of welfare breaches, ecological degradation and pathogen spillover in livestock–wildlife–human interfaces—especially in marginalized areas—exposes systemic neglect of public health. One Welfare provides a vital integrative response, bridging the agendas of animal welfare, One Health, and Planetary Health. However, Uruguay's refusal to legally recognize animal sentience perpetuates a property-based framework that is at odds with EU and WOAH standards, despite scientific consensus on animals' cognitive, emotional, and social capacities [4]. This gap demands not only legal reform but an ethical shift aligned with ecological and public health imperatives. Stray dogs alone have generated multi-million-dollar livestock losses and psychosocial harm [26,48,51]. At the same time, zoonotic threats from bats, boars, roaming horses, and cats reveal the urgent need for multi-sectoral, ethically grounded governance [72,89,93].

**Fig. 6:**
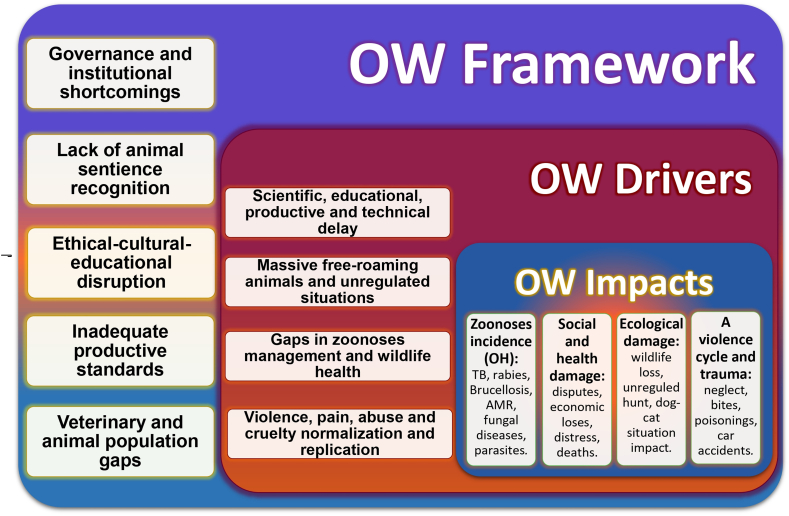
Conceptual framework linking One Welfare (OW) governance and institutional shortcomings to systemic drivers and public health impacts. Abbreviations: OH, One Health; TB, tuberculosis; AMR, antimicrobial resistance.

Fig. 6 conceptualizes these interlinked failures, connecting governance and cultural shortcomings with cascading drivers—free-roaming animals, weak zoonotic disease management, and normalized cruelty—that translate into measurable One Welfare impacts across species.

Thousands of violations reported in the media—from poisonings and biopark neglect to mass heat-stress deaths—remain excluded from institutional science, creating an evidentiary gap that obscures risks and weakens systemic responses. Mapping these silences is crucial for strengthening One Welfare and fostering participatory governance.

One Welfare must therefore move beyond concept to practice: a systems-thinking tool embedded in health, environmental, and welfare policies to build resilience in the face of planetary crises. These challenges extend beyond Uruguay, demanding integrative research methods—such as index analysis, reviews, and digital epidemiology—to support global reform in line with the objectives of One Welfare, One Health, and Planetary Health.

Ultimately, beyond laws and data, an ethical void persists in the vanished animals of forests, streets and homes—never counted, never saved. Their absence marks a national conscience, reminding us that true character lies not in claims but in tolerations. Each lost life, animal or human, scars both land and memory. From this silence rises a call: to rebuild a future rooted in empathy, justice, and multi-species solidarity.

## 5 Conclusion

The Uruguayan case illustrates how outdated, human-centric health ideas fail to address systemic zoonotic risks in multi-species contexts. Institutions often overlook animal sentience, ecological fragility, and social trauma, resulting in both epidemiological and ethical failures. A shift to systems thinking is urgently needed, with One Welfare at the core of public health resilience in the Anthropocene, offering a clear diagnostic and strategic lens—especially in places where zoonoses emerge within informal, poorly regulated human–animal–environment interfaces. These governance gaps are likely not unique to Uruguay, and we urge researchers and policymakers to use assessments that combine welfare indicators, zoonotic risk mapping, and socio-environmental analysis. This approach can help establish a shared One Health reform agenda. The One Health–One Welfare nexus is not abstract: it is a vital frontier for ethical, sustainable, and coordinated public health systems. Public health must go beyond traditional metrics and adopt a multi-species justice approach. One Welfare is no longer merely a moral idea, but an urgent policy tool for human, animal, and planetary resilience. To implement it, legal, educational, and institutional reforms are needed—grounded in local realities and driven by cultural change. Uruguay and similar nations must bridge the gap between progressive discourse and structural inertia. In our era of climate disruption, social vulnerability, and zoonotic uncertainty, embedding One Welfare is not optional: it is essential for justice, sustainability, and collective survival.

## CRediT authorship contribution statement

**Bernabé Vidal:** Writing – review & editing, Writing – original draft, Investigation, Funding acquisition, Data curation, Conceptualization. **Lorenzo Verger:** Writing – review & editing, Validation, Investigation, Data curation. **Gustavo J. Nagy:** Writing – review & editing, Supervision, Investigation, Data curation, Conceptualization.

## Declaration of generative AI and AI-assisted technologies in the writing process

During the preparation of this work, the authors used ChatGPT-4, O1 preview in order to support the writing process (improving readability). After using this tool/service, the authors reviewed and edited the content as needed and take full responsibility for the content of the published article.

## Funding

This work was supported by the ANII (*Agencia Nacional de Investigación e Innovación*) [grant number POS_NAC_2023_1_178487], and the “*Programa de Posgrado en Ciencias Ambientales*,” *Facultad de Ciencias*, *Universidad de la República*, Uruguay.

## Conflict of interest

The authors declare that they have **no known competing financial interests or personal relationships** that could have appeared to influence the work reported in this paper.
