# Supplementary material for: Exploring the One Health–One Welfare nexus and zoonoses
Source: Sci One Health. 2025 Oct 13;4:100128. doi: 10.1016/j.soh.2025.100128 (PMC12630043; doi:10.1016/j.soh.2025.100128)
Supplement: Multimedia component 1 [file mmc1.docx]

**Supplementary material
Full tables and Documents excluded from main body results (2015-2025).


Table 1.** All relevant documents from 2015-2019 in Uruguay.

| **Topic** | **Summary of published documents** |
| --- | --- |
| **Animal Welfare and Veterinary profession** | Among 112 surveyed veterinarians working in cattle or meat production, a majority expressed a desire for more training on animal welfare (AW), and 85% supported AW in curricula [1]. Another study found that most of the 388 surveyed vets had some AW training; 95% supported AW in curricula, but over half lacked awareness of the regional strategy. More formal education is needed [2].  A survey of 100 clinics found that veterinarians believed male dogs were more aggressive, particularly those of the Pit bull, German Shepherd, and Cimarron breeds. Cimarrón was ranked the highest, raising concerns about welfare and safety for certain breeds [3].  Compared two egg farms (large and family-based) using WQ® methods. Both met basic AW standards for space, dust-bathing, and perches. Minor issues included feather picking, heat stress, and insufficient activities for the hens. The study calls for more data and greater awareness, especially since both farms were cage-free [4].  Sport hunting sparked national debate in 2017, revealing tensions between conservation (“Uruguay Natural”) and production (“Uruguay Productivo”). Issues include native–exotic species conflicts, ethical rejection of suffering, and hunters acting as poorly regulated, state-backed pest managers, with frequent injuries and abandonment of hunting dogs [5]. |
| **Animal Transport and slaughter** | Shipments to slaughterhouses showed frequent carcass bruising despite common driver welfare training. Electric prods were still widely used. Studies recommend better trucks, improved handling, staff training, and enhanced inspections. Tools like PROGAT® and flags reduced bruises and meat loss [6,7,8]. Bruises affected 44.4% of cow carcasses, mostly minor and more frequent in females and older animals, indicating the need for improved transport practices [9]. Analysis of 15,157 carcasses from 13 slaughterhouses found 60% bruised, mainly minor on the rump, with an average meat loss of 899 g per animal due to poor transport and handling, calling for better facilities and training [10].  Horse stunning was successful on the first attempt in 97% of cases, demonstrating overall effectiveness. However, delays in bleeding and a 9.91% rate of head lifting after stunning at one plant highlight gaps needing further research, clearer regulations, and better staff training [11]. |
| **Bovine-ovine production** | A survey of 33 vets found that disbudding and dehorning are common, but 79% do not use pain control. Most procedures were done by non-vets; only 21% used pain relief. This signals a need for stronger ethics [12]. Of the 38 surveyed vets, 94.7% remove horns from calves; 80.6% skip pain relief, mostly due to time and cost constraints. Only 19.4% use pain relief. Many underestimate the need for pain control. Needed: pain relief, rules, and training [13].  A feedlot survey found main causes of death were digestive problems (44%), hoof and foot infections (25%), urinary issues (20%), and respiratory problems (10%). Sudden diet shifts from grass to grain triggered digestive losses, while injuries exposed facility and handling flaws. The study recommends smoother dietary transitions and better facility designs [14].  A study found that dairy cows on pasture displayed more natural behaviour than those in indoor stalls, although stall design greatly influenced comfort. Pasture access allowed the most natural activity, suggesting potential welfare benefits that require further research [15]. Hoof disease data in dairy cows shifted from mainly infectious causes (2001–2004) to injury or diet-related issues by 2016, with white line disease making up 67.61%; smaller herds had more cases, highlighting the need for regular checks [16]. A 2015 dairy farm case reported hoof problems (4.93%), including heel damage, overgrown toes, white line splits, and skin swelling, varying by age and lactation stage. Recommendations include improved facilities, regular trimming, foot baths, and greater care for younger cows [17]. |

**Table 2.** All relevant documents from 2020 to 2025 in Uruguay.

| **Topic** | **Summary of Published documents** |
| --- | --- |
| Animal Welfare | A review summarises the evolution of animal welfare in Uruguay and globally, its links to One Health/One Welfare, differences from animal rights, consumer influence, welfare indicators, frequent issues (e.g., transport hematomas), and key institutional milestones [18]. A legal comparison shows the need to strengthen welfare laws and enforce animal legal personhood, hindered by limited resources [19].  Uruguay has contributed little to animal welfare research, with ruminant studies starting late and remaining scarce. In 2018, it produced only 0.35% of global bovine and 0.58% of ovine publications, about 20 years behind other regions, weakening its political and ethical standing internationally [20].  A survey of veterinarians showed perioperative pain in companion animals is often undertreated, with reliance on weak opioids or NSAIDs due to cost, regulation, and limited training. Thirty-eight percent felt unprepared, revealing a gap between pain severity and treatment, and the need for better education and access to analgesics [21].  Endurance racehorses (2007–2018) had a fatality rate of 5.9 per 1,000 starts, higher in short races (sudden death) and long races (metabolic causes) [22]. A 2011–2017 study on thoroughbreds found 0.42 musculoskeletal injuries per 1,000 starts and 0.059 per 100 horse months in training. Despite lower rates compared to other countries, persistent welfare concerns demand closer monitoring [23].   Conflicts with free-ranging dogs persist: the Rural Code permits killing to protect livestock, while the Animal Welfare Law prohibits cruelty [24]. Attacks affected 49% of surveyed farmers, causing severe distress (p < 0.01) [25]. Broader issues include livestock losses, weak governance, and education gaps, prompting calls for legislation, sociocultural change, and interdisciplinary action [26]. In 2018–2019, 848 reports recorded 7,163 sheep harmed by stray dogs, revealing underreporting, uneven distribution, and institutional gaps, urging better data and evidence-based solutions [27].  Hunting dog conflicts in Uruguay highlight tensions between hunters, who consider them partners, and activists, who view the practice as cruel. Legal gaps leave dogs unregulated and boars labelled as pests, while fragmented institutions hinder integrating welfare, conservation, and governance [28]. Broader tensions over wild boar hunting mirror these divides, with unclear ethics and policy gaps prompting calls for stronger frameworks and improved institutional coordination [29].  A survey of 100 Montevideo veterinary clinics found canine aggression mostly managed through obedience training (91%), psychotropics (47%), euthanasia (28%), and castration (7%). Few referred cases (50%) or requested diagnostics (14%), highlighting the need to integrate behavioural training into veterinary curricula [30]. Canine aggression is the most frequent behavioural issue, linked to abandonment, euthanasia, livestock attacks, and public health risks, with the Cimarrón Uruguayo ranking highest in reported cases [31].  Evaluated enclosure design for Geoffroy's cat. Found limited activity, space-use bias, and 79% indicator deficits (Ackonc-AWA). Recommends redesign and broader use of the methodology for other species [32]. |
| Animal Transport  and Slaughter | Monitoring 12 cattle shipments revealed mostly adequate trucks, but persistent welfare problems: 77% of carcasses had subcutaneous hematomas, stress markers were high, and poor handling occurred (92 blows, slippery unloading, reckless driving). The study recommends improving transport, infrastructure, and handling [33]. In 123 additional journeys (4,062 cattle), despite 78% of drivers being trained, 52.85% used electric prods and 31.7% of animals were hit, with 40.2% of carcasses showing bruises (grade 1–2). Persistent issues highlight the need for stricter audits, improved training, and reforms [34].  Uruguayan research on bovine pre-slaughter bruises found most tools ineffective for dating lesions, but infrared thermography could detect bruises older than 12 hours, aiding welfare and traceability [35]. A separate study on 60 pasture-finished steers showed transport caused physical but not psychological stress, while a longer lairage (15 h) improved carcass pH and recovery. Calmer animals performed better, highlighting lairage time and temperament as key factors for welfare, meat quality, and policy design [36].  Horse transport to slaughterhouses in Argentina and Uruguay revealed welfare issues, with temperatures and THI exceeding thermoneutral limits in six cases. Aggression varied by compartment, and flaws in infrastructure and separation were noted. The study recommends better ramp design, driver training, and stocking practices to improve equine transport welfare [37]. |
| Bovine-ovine Production | In feedlots, shade improved behaviour, rumen pH, feed efficiency, daily gain (+13%), and carcass weight. Deaths occurred under extreme heat without mitigation, showing the need for shade, water, dietary changes, and special care [38]. Trials in Salto using the Heat Load Index found shade improved weight gain during mild or no heatwaves but was insufficient in strong ones, stressing the importance of natural shade for welfare and productivity in rangeland systems [39].  Evaluations in 15 dairy farms revealed welfare issues, including lameness, poor paths, lack of shade and water, with elevated cortisol in lame cows [40]. In 12 herds, annual culling reached 23.1%, mainly due to reproductive failure (29.3%) and mastitis (25.9%) [41]. Monitoring 5,375 cows across 13 herds showed high early-lactation disease rates—mastitis (27.6%), lameness (5.0%), RP-metritis (4.4%)—especially in multiparous animals [42]. A survey of 225 herds found 15.2% pre-weaning calf mortality linked to poor colostrum use, hygiene gaps, and limited veterinary support (38.7%) [43].  A survey of 81 producers in Cerro Largo revealed major welfare gaps: late castration without analgesia (84%), use of electric prods (28%), limited shade and water, and lack of empathy. While 60% of producers had training, only 20% of workers did. The study urges improved infrastructure, worker education, and supportive public policies [44].  A study of 100 ovine abortion cases (2015–2021) found *Toxoplasma gondii*, *Campylobacter fetus*, and dystocia as the main causes, with necropsies showing fetal suffering. Many cases were preventable, raising welfare and zoonotic concerns and highlighting the value of abortion monitoring for reproduction and public health [45]. BVDV was also detected, with some unknown or non-infectious causes. Limited vaccine access and diagnostic gaps stress the need for improved reproductive health strategies [46]. |

**Table 3. Other documents included.**

| Results |
| --- |

| A thesis-based assessment of a capybara farm in Canelones revealed generally acceptable welfare but with deficiencies in water access, shelter, and feeding. Institutional gaps—such as the lack of legal slaughterhouses and inadequate state support—encourage producers to adopt informal practices, thereby weakening ethical standards and compromising the traceability of products [47]. |
| --- |
| Social rank in pampas deer females influenced flight distance and maternal response to fawn handling. High-ranked hinds showed greater avoidance of humans and maintained more distance from their fawns. Findings suggest adapting handling strategies based on social hierarchy in reserves to reduce stress and improve management in semi-captive populations [48]. |
| Compared 100% natural grassland (NG) with partially forested land (FL, 60% *Pinus taeda*). FL had better forage protein and lower temps, improving grazing and daily gains. Tree integration enhanced thermal comfort, nutrition, and productivity—supporting welfare and sustainability [49]. |
| A 2017 survey (n = 663) found strong support for controlling invasive species but ethical resistance to lethal methods, especially for charismatic animals such as the axis deer. While pest species faced less opposition, animal lovers showed high rejection of any killing, revealing a divide between the conservation and management sectors. The study advocates for education, legal reform, and inclusive strategies that address biodiversity loss in the context of animal welfare and suffering [50]. |
| Silvopastoral systems (SPS) are a viable option for traditional livestock producers. They allow diversification without compromising productivity or animal welfare. SPS offers economic resilience from livestock and timber income. It also enriches environments and promotes natural cattle behaviours. SPS can be locally adapted to enhance sustainability [51]. |
| One hundred thirty beef cattle were monitored in silvopastoral and open pasture systems. No welfare issues or performance differences were found. Silvopastoral systems offer environmental enrichment and an additional income from timber, supporting sustainability while maintaining animal welfare [52]. |
| Confinement reduced rumination and lying behaviour despite short-term gains in blood markers and milk yield. Milk from confined cows showed lower concentrations of CLA and omega-3 fatty acids. Night grazing preserved welfare and improved fatty acid profile. Mixed systems outperformed confinement in terms of behaviour and nutritional quality, especially when open-air enclosures and pasture access were maintained [53]. |
| An experimental study tested four fattening systems in Hereford steers. High-concentrate diets boosted growth but worsened welfare—especially with ad libitum feeding—due to increased stress, disease, and mortality. Pasture-based systems improved welfare and meat tenderness. Calm animals had better productivity and carcass quality [54]. |
| Integrating native forests into grazing systems improves welfare by providing shade, better forage, and reducing heat stress. It is essential to promote research, planning, and national recognition of the benefits of forests and wildlife [55]. |
| There is a rising concern over heat stress in Uruguayan cattle systems. High temperatures, humidity, and solar radiation impact welfare and productivity, reducing feed intake and fertility and causing behavioural changes or even death. It emphasises ethical responsibilities, climate change implications, and mitigation strategies such as shade, water conservation, and improved management. Heat stress is framed as both an animal welfare and market issue [56]. |
| An observational study compared dairy cows lying in postures on pasture (Uruguay/Netherlands) versus in cubicles (Netherlands), finding that indoor housing restricts natural behaviours. While cubicle design, bedding, and dividers can affect comfort, these features do not offer the same freedom of movement as pasture. The impact on welfare is not definitively established in the study, but it suggests that systems allowing more behavioural flexibility may support better welfare outcomes [57]. |
| The analysis of 383 horses during 720 “jineteada” rides revealed that 9.5% of the rides were completed, 3.19% required treatment, and there were no deaths or fractures. Natural behaviour was observed. The transport was not ideal, but the handling was acceptable. Urges the development of unified national protocols and further studies to compare the situation with other festivals in the country [58]. |
| Compared cows raised on natural pasture to those in forested areas. Both tree-pasture and open pasture yielded similar animal welfare and gains, but tree-pasture also provided additional benefits, such as shade and wood [59]. |

**Supplementary References:**

1. S.M. Huertas, D. César, J. Piaggio, A. Gil, Evaluation of Education of Veterinarians in Animal Welfare Issues, Journal of Agricultural Science and Technology, 5(2) (2015 a.) 136-140. <https://www.researchgate.net/publication/280153574_Evaluation_of_Education_of_Veterinarians_in_Animal_Welfare_Issues>
2. M.M. Prieto-Laport, P.E. Bobadilla-Robledo, S.M. Huertas-Canén, Animal Welfare in the Uruguayan Veterinary Profession Field, Journal of Agricultural Science and Technology, A7 (2017) 357-362. <https://bienestaranimal.org.uy/wp-content/uploads/2022/06/AW-in-Vet-profes_Prieto_bobadilla_Huertas.pdf>
3. P. Ruiz-Santos, M. Belino, R. Rijo, J. Piaggi, J.P. Damián, Clinical Veterinarians'' Perceptions about Sexes and Breeds of Dogs Involved in Aggressiveness in Montevideo, Uruguay, Society & Animals, 27(2) (2018). <https://brill.com/view/journals/soan/28/2/article-p191_6.xml>
4. N.S. Gonzalez-Riccio, C.M. Perez-Mendez, Evaluación del bienestar animal basado en el protocolo Welfare quality ® para ponedoras de la especie *Gallus gallus* *domesticus* en piso en algunas granjas de Uruguay. Degree Thesis, Facultad de Veterinaria, Universidad de la República, Uruguay. (2017). https://www.colibri.udelar.edu.uy/jspui/bitstream/20.500.12008/24970/1/FV-32946.pdf
5. J.M. Dabezies, Discursos y tensiones entre caza, conservación y derechos de los animales en Uruguay, Etnobiología, 17(2) (2019) 11-24. https://revistaetnobiologia.mx/index.php/etno/article/view/106/104
6. S.J. Gibernau-Arredondo, M.A. Resio-Laxalde, Estudio de algunas características del transporte de bovinos y su relación con indicadores de bienestar animal en Uruguay. Degree Thesis, Facultad de Veterinaria, Universidad de la República, Uruguay. (2018). <https://www.colibri.udelar.edu.uy/jspui/bitstream/20.500.12008/25138/1/FV-33343.pdf>
7. S.M. Huertas, P.E. Bobadilla, M. Prieto, F. Villa, J. Lestido, Evaluation of a protection system for livestock during transport: PROGAT® from the animal welfare point of view, Biomedical Journal of Scientific & Technical Research, 6(4) (2018). <https://bienestaranimal.org.uy/wp-content/uploads/2022/06/Progat.pdf>
8. S.M. Huertas, R.E.A.M. Kempener, F.J.C.M. Van Eerdenburg, Relationship between Methods of Loading and Unloading, Carcass Bruising, and Animal Welfare in the Transportation of Extensively Reared Beef Cattle. Animals*,* 8(7) (2018 b.) 119. https://www.mdpi.com/2076-2615/8/7/119
9. G. Crosi, M. Prado, S. Huertas, J. Imelio, J. Piaggio, A. Gil, Estudio observacional sobre presencia y caracterización de hematomas en carcasas vacunas de Uruguay, Salud y Tecnología Veterinaria, 3(2) (2015) 41-50. <https://revistas.upch.edu.pe/index.php/STV/article/view/2824>
10. S.M. Huertas, F. Van Eerdenburg, A. Gil, J. Piaggio, Prevalence of carcass bruises as an indicator of welfare in beef cattle and the relation to the economic impact, Veterinary Medicine and Science, 1 (2015 B.) 9-15. <https://onlinelibrary.wiley.com/doi/pdfdirect/10.1002/vms3.2>
11. M.A. Loureiro-Suárez, M.S. Sosa-Fulquet, Efectividad del noqueo como indicador de bienestar animal en plantas de faena de equinos de Uruguay. Degree Thesis, Facultad de Veterinaria, UDELAR, Uruguay. (2015). https://www.colibri.udelar.edu.uy/jspui/bitstream/20.500.12008/10274/1/FV-31533.pdf
12. R.D. Caffarena, F. Riet-Correa, .F. Giannitti, Uso de protocolos de manejo del dolor durante el desbotonamiento y descorne de las terneras de tambo: ¿qué tan común es en Uruguay y Argentina?, Jornadas Uruguayas de Buiatría (2017). <https://bibliotecadigital.fvet.edu.uy/bitstream/handle/123456789/2864/JB2017_217-219.pdf?sequence=1&isAllowed=y>
13. R.D. Caffarena, F. Riet-Correa, F. Giannitti, Uso de prácticas de manejo del dolor durante el desbotonamiento y descorne de las terneras de tambo: un estudio piloto en Uruguay y Argentina, Veterinaria (Montev.), 54(210) (2018). http://www.scielo.edu.uy/scielo.php?pid=S1688-48092018000200022&script=sci_arttext
14. G. Banchero, D. Chalkling, A. Mederos, Relevamiento de problemas sanitarios y de manejo durante la terminación en bovinos en sistemas de confinamiento en Uruguay, Veterinaria (Montevideo) 52(202) (2016) 1. <http://scielo.edu.uy/scielo.php?script=sci_arttext&pid=S1688-48092016000200001>
15. E. van Erp-van der Kooij, O. Almalik, D. Cavestany, J. Roelofs, Lying Postures of Dairy Cows in Cubicles and on Pasture, Animals, 9(4) (2019) 183. <https://www.researchgate.net/publication/332584332_Lying_Postures_of_Dairy_Cows_in_Cubicles_and_on_Pasture>
16. L.C. González-Pulchavert, M. Castrillón-Amaya, Problemas podales en bovinos de establecimientos lecheros de los departamentos de Florida y San José. Degree Thesis, Facultad de Veterinaria, Universidad de la República, Uruguay. (2017) [https://www.colibri.udelar.edu.uy/jspui/bitstream/20.500.12008/24965/1/FV 32842.pdf](https://www.colibri.udelar.edu.uy/jspui/bitstream/20.500.12008/24965/1/FV%2032842.pdf)
17. J.N. Jourdan-Pita, G.A. Rivera-Pacheco, Estudio observacional de afecciones podales en un tambo comercial en el departamento de Colonia. Degree Thesis, Facultad de Veterinaria, Universidad de la República, Uruguay. (2019). https://www.colibri.udelar.edu.uy/jspui/bitstream/20.500.12008/25749/1/FV-34063.pdf
18. S.M. Huertas, Bienestar Animal: una preocupación mundial, ¿qué hemos hecho en el Uruguay? 50L Jornadas Uruguayas de Buiatria. 8-9 junio, 2023. <https://bibliotecadigital.fvet.edu.uy/bitstream/handle/123456789/3290/JB2023_171-180.pdf?sequence=1&isAllowed=y>
19. S.M. Armand-Ugón, A. Montero-Susalla, La protección de los animales a la luz del derecho: ¿tienen derechos los animales?, Rev. Derecho (2023), 27 e2925. <http://www.scielo.edu.uy/scielo.php?script=sci_arttext&pid=S2393-61932023000101203>
20. R. Ungerfeld, Bienestar en animales de producción: la investigación nacional, ¿acompasa las exigencias internacionales en el tema?, Veterinaria (Motnev.), 56(213) (2020). http://www.scielo.edu.uy/pdf/vet/v56n213/1688-4809-vet-56-213-e201.pdf
21. J.G. Badía, G.L. Figueiro, L. Recchi, V. Machín, E. Rossini, N. Crosignani, Current attitudes towards the use of perioperative analgesics in small animals by Uruguayan veterinarians, Austral. J. Vet. Sci., 54(3) (2022). https://www.scielo.cl/scielo.php?script=sci_arttext&pid=S0719-81322022000300127&lang=pt
22. G. Brito, J.P. Damián, G. Suárez, et al., Characterisation of Raid Hipico Uruguayo Competencies by Ride Type: Causes of Death and Risk Factors, Animals (Basel), 13(10) (2023). <https://pubmed.ncbi.nlm.nih.gov/37238032/>
23. N.H. Bimson, A.V. Morrice-West, A.S.M. Wong, et al., Catastrophic Musculoskeletal Injuries in Thoroughbred Racehorses in Uruguay, 2011-2017, J Equine Vet Sci, Oct (2022);117:104074. https://pubmed.ncbi.nlm.nih.gov/35820498/
24. B. Puppo-Hatchondo, L.A. Tealde-Zapata, Perros y Ovinos: Conflictos y armonía en su régimen de protección jurídica, Aportes al VII Congreso Nacional de Derecho Agrario Provincial, Universidad Nacional de la Plata, Argentina. <https://sedici.unlp.edu.ar/bitstream/handle/10915/176093/Documento_completo.pdf?sequence=1>.
25. P. Ruiz, G. Benítez, P.E. Bobadilla, et al., Do predator attacks on productive species and the respective economic losses influence the psychological distress of farmers in Uruguay? A cross sectorial study, Rural and remote health, (2024) 24:7614. https://search.informit.org/doi/pdf/10.3316/informit.T20240425000132015566592 44
26. L. Poliak-Almeida, J.M. Dabezies, El problema no son los perros. Una mirada antropológica al debate de los perros sueltos en Uruguay, Farol - Revista de estudos organizacionais e sociedade, 11(30) (2024) 175-212. https://www.researchgate.net/profile/Juan-Dabezies/publication/380600454_El_problema_no_son_los_perros_Una_mirada_antropologica_en_torno_al_debate_de_los_perros_sueltos_en_Uruguay/links/664550ba0b0d28457436e081/El-problema-no-son-los-perros-Una-mirada-antropologica-en-torno-al-debate-de-los-perros-sueltos-en-Uruguay.pdf
27. E. Barcos-Silveira, Ataques de perros a ovinos: estimación de pérdidas económicas en Uruguay. Degree Thesis, Facultad de Veterinaria, UDELAR, Uruguay. (2023) <https://www.colibri.udelar.edu.uy/jspui/bitstream/20.500.12008/42127/1/FV-36050.pdf>
28. L. Poliak, J.M. Davezies, Enredos perrunos: el perro de caza mayor en Uruguay desde diferentes colectivos sociales, Tabula Rasa, 40 (2022) 99-122. <https://revistas.unicolmayor.edu.co/index.php/tabularasa/article/view/1990>
29. L. Poliak-Almeida, (Des)encuentros interespecies Tensiones en torno a la caza del jabalí en Uruguay, Revista Andaluza de Antropología*,* 21 (2021) 45-61. <https://dialnet.unirioja.es/servlet/articulo?codigo=8282497>
30. P. Ruiz-Santos, G. Suárez, J.P. Damián, Abordajes y tratamientos de la agresividad en perros en clínicas veterinarias de Montevideo (Uruguay), Rev. Arg. Cs. Comp., 14 (2) (2022). https://www.scielo.org.ar/scielo.php?pid=S1852-42062022000200127&script=sci_arttext&tlng=es
31. J.P. Damián, Aggressiveness in dogs, a problem in health and Welfare*,* Revista – Facultad Nacional de Agronomía Medellín, 74 Suplemento, S44-S47 (2022). <https://www.cabidigitallibrary.org/doi/full/10.5555/20220176838>
32. M. Lauria, S. Corte, D. Racciatti, Evaluación y diseño de recintos centrados en el bienestar animal: estudio de caso en Leopardus geoffroyi, Boletín de la Sociedad zoológica del Uruguay, 34(1) (2025). https://szu.org.uy/journal/index.php/Bol_SZU/article/view/325
33. I. Bonino-Legunas, M. Bove-Callero, M. Antúnez, X. Lagomarsino, Evaluation of the relationship between transport handling and unloading of beef cattle, animal welfare and meat quality, Tellus, 3(1) (2023) 40-63. <https://www.fca-ude.edu.uy/tellus/pdfs/Tellus-N-003-2023-03.pdf#page=41>
34. C.D. Hernández-Baldivieso, M.V. Martínez-Domínguez, Caracterización del transporte de bovinos a plantas de Faena y su veón con la prevalencia de hematomas (machucones) en las carcasas. Degree Thesis, Facultad de Veterinaria, Universidad de la República, Uruguay. (2023). <https://www.colibri.udelar.edu.uy/jspui/bitstream/20.500.12008/42363/1/FV-36096.pdf>
35. M, del Campo-Saravia, Evaluación de herramientas para la determinación de la antigüedad de los hematomas ocurridos en las últimas etapas previas a la faena en bovinos para carne. Degree Thesis, Facultad de Veterinaria, Universidad de la República, Uruguay. (2023). <https://www.colibri.udelar.edu.uy/jspui/bitstream/20.500.12008/42339/5/del%20Campo%20Manuela.pdf>
36. M. Del Campo-Gigena, J.M. Soares De Lima, G. Brito, X. Manteca, P. Hernández, F. Montossi, Effect of finishing diet and lairage time on steers welfare in Uruguay, Animals, 11(5) (2021 b.) 1329. https://www.mdpi.com/2076-2615/11/5/1329
37. B. Nivelle, L. Vermeulen, S. Van Beirendonck, et al., Horse Transport to Three South American Horse Slaughterhouses: A Descriptive Study. Animals, 10(4) (2020) 602. https://www.mdpi.com/2076-2615/10/4/602
38. M.E.A. Canozzi, J. Clariget, G. Roig, et al., ¿Existe estrés por calor en ganado a corral en uruguay? Resultados y recomendaciones, Revista INIA, Septiembre (2021) 29-32. <https://ainfo.inia.uy/digital/bitstream/item/15999/1/Revista-INIA-66-Setiembre-2021-9.pdf>
39. C. Saravia, E. van Lier, C. Munka et al., Trees on rangelands can attenuate the negative effect of heat waves on Hereford heifers'heifers' productivity, Agroforestry Systems, 98 (2024) 2431-2448. <https://link.springer.com/article/10.1007/s10457-024-01039-x>
40. V.R. Boroski-Reske, S.M. Martino-Quartara, M.M. Prieto-Laport, Caracterización de vacas lecheras a través de indicadores de bienestar animal y su relación con prácticas de manejo, infraestructura y medio ambiente en predios lecheros del Uruguay. Degree Thesis, Facultad de Veterinaria, Universidad de la República, Uruguay. (2023). https://www.colibri.udelar.edu.uy/jspui/bitstream/20.500.12008/19381/6/Boroski%2c%20V..pdf
41. B. Doncel-Díaz, S. Fariña, R.D. Caffarena, F. Giannitti, F. Riet-Correa, Cow Culling Rates and Causes in 12 Pasture-Based Dairy Herds in Southern Uruguay, a Pilot Study, Dairy (Basel), 6(1) (2025). <https://www.proquest.com/openview/a5214a5702fabb161a9e06e5c56db2c8/1?pq-origsite=gscholar&cbl=5046907>
42. I. Cruz, I. Pereira, G. Ruprechter, J. Barca, A. Meikle, A. Larriestra, Clinical disease incidence during early lactation, risk factors and association with fertility and culling in grazing dairy cows in Uruguay, Preventive Veterinary Medicine, 191 (2021) 105359. https://www.sciencedirect.com/science/article/abs/pii/S0167587721001033
43. C.O. Schild, R.D. Caffarena, A. Gil, et al., A survey of management practices that influence calf welfare and an estimation of the annual calf mortality risk in pastured dairy herds in Uruguay. Journal of Dairy Science, (2020) 9418-9429. <https://www.sciencedirect.com/science/article/pii/S0022030220305968>
44. T. Apolo, F. Guerrero, B. Litta, R. Gómez-Miller, M. del Campo. Valoración de prácticas vinculadas al bienestar animal en productores ganaderos extensivos del departamento de Cerro Largo, Revista Tellus, mayo 2025(7): 59-78. <https://www.fca-ude.edu.uy/tellus/pdfs/Tellus-N-007-2025-05.pdf#page=60>
45. M.A. Dorsch, M.E. Francia, L.R. Tanta, et al., Diagnostic Investigation of 100 Cases of Abortion in Sheep in Uruguay: 2015–2021, Front. Vet. Sci*.,* 9 – 2022. <https://www.frontiersin.org/journals/veterinary-science/articles/10.3389/fvets.2022.904786/full>
46. F. Giannitti, M.E. Francia, L. Tana, et al., Causas de aborto en ovinos de Uruguay: 100 casos, 2015-2021, Revista INIA*,* 70 (2022) 18-22*.* https://ainfo.inia.uy/digital/bitstream/item/16786/1/Revista-INIA-70-setiembre-2022-06.pdf
47. M. Minteguiaga, Diagnóstico de bienestar animal en un criadero intensivo de carpinchos (Hydrochoerus hydrochaeris) en Salto, Uruguay, Spei Domus. 2015; 11(23) (2015) 47-54. <https://www.academia.edu/28728646/Diagn%C3%B3stico_de_bienestar_animal_en_un_criadero_intensivo_de_carpinchos_Hydrochoerus_hydrochaeris_en_Salto_Uruguay>
48. R. Ungerfeld, J.T. Morales-Piñeyrúa, A. Freitas-De-Melo, Flight distance and reaction to fawn handling are greater in high- than in low-ranked pampas deer (Ozotoceros bezoarticus) females. Animal Welfare*,* 24 (2015) 391-397. <https://www.researchgate.net/profile/Aline-Freitas-De-Melo/publication/283225576_Flight_distance_and_reaction_to_fawn_handling_are_greater_in_high-than_in_low-ranked_pampas_deer_Ozotoceros_bezoarticus_females/links/562ebfe508ae518e34838432/Flight-distance-and-reaction-to-fawn-handling-are-greater-in-high-than-in-low-ranked-pampas-deer-Ozotoceros-bezoarticus-females.pdf>
49. J.K. Fedrigo, R. Santa Cruz, V. Benítez, et al., Dynamics of forage mass, air temperature and animal performance in a silvopastoral system of Uruguay. Agroforest. Syst*.,* 93 (2018) 2197-2204. <https://link.springer.com/article/10.1007/s10457-018-0335-2>
50. G. Laufer, Avances de estudios en Uruguay: el dilema del control y erradicación de las especies exóticas invasoras ante los requerimientos de la opinión pública, in: F. Texeira de Melo (Ed.), Experimentación con animales no tradicionales en Uruguay, CHEA-UdelaR, 2019, 225-238. <https://www.researchgate.net/publication/337901531_Avances_de_estudios_en_Uruguay_el_dilema_del_control_y_erradicacion_de_las_especies_exoticas_invasoras_ante_los_requerimientos_de_la_opinion_publica>
51. INIA, Evaluación de la sustentabilidad de los sistemas productivos silvopastoriles y sistemas forestales existentes en el país y su relación con la producción de bovinos de carne, Serie FPTA-INIA, 87 (2020). <https://ainfo.inia.uy/digital/bitstream/item/14475/1/Inia-Fpta-87-proyecto-311-2020.pdf#page=57>
52. S.M. Huertas, P.E. Bobadilla, I. Alcántara, E. Akkermans, F.J.C.M. Van Eerdenburg, Benefits of Silvopastoral Systems for Keeping Beef Cattle. Animals, 11(4) (2021) 992. <https://www.mdpi.com/2076-2615/11/4/992>
53. L. Grille-Pees, Manejos de alimentación y ambiente en los sistemas lecheros de base pastoril en vacas con partos de primavera: impactos en el bienestar animal, producción, composición y perfil de ácidos grasos en la leche y queso. Degree Thesis, Facultad de Veterinaria, Universidad de la República, Uruguay. (2023). <https://www.colibri.udelar.edu.uy/jspui/bitstream/20.500.12008/42435/1/FV-36108.pdf>
54. M. del Campo, X. Manteca, J.M. Soares De Lima, G. Brito, P. Hernández, C. Sañudo, F. Montossi, Effect of Different Finishing Strategies and Steer Temperament on Animal Welfare and Instrumental Meat Tenderness. Animals, 11(3) (2021 a.) 859. <https://www.mdpi.com/2076-2615/11/3/859>
55. F. Soares De Lima, M. Pereira-Machin, Estado de conocimiento sobre los beneficios del bosque nativo y las especies nativas sobre la producción ganadera, Seria Técnica INIA 261, Enero 2022. <https://www.inia.uy/sites/default/files/publications/2024-10/st-261-2022.pdf#page=18>
56. V. Herrera-Costabel, Efecto del estrés calórico en el bienestar animal y la producción, Revista Plan Agropecuario, 176 (2020) 25-27. https://www.planagropecuario.org.uy/uploads/magazines/articles/191_2972.pdf
57. E. van Erp-van der Kooij, O. Almalik, D. Cavestany, J. Roelofs, Lying Postures of Dairy Cows in Cubicles and on Pasture, Animals, 9(4) (2019) 183. <https://www.researchgate.net/publication/332584332_Lying_Postures_of_Dairy_Cows_in_Cubicles_and_on_Pasture>
58. C.A. Iribarren-Pose, L.M. Navarro-De León, Descripción de la semana criolla, tomando en cuenta “indicadores” de bienestar animal. Degree Thesis, Facultad de Veterinaria, Universidad de la República, Uruguay. (2020). <https://www.colibri.udelar.edu.uy/jspui/bitstream/20.500.12008/28982/1/FV-34465.pdf>
59. S.M. Huertas, P.E. Bobadilla, H.J. Bueno, D. Cesar, J.M. Piaggio, A.D. Gil, Environmental Conditions in a Temperate Weather Silvopastoral System vs. Natural Grassland and their Impact on Animal Beef Production, Biomed J Sci & Tech Res, 3(2) (2018). <https://www.researchgate.net/profile/Stella-Huertas/publication/326474893_Relationship_between_Methods_of_Loading_and_Unloading_Carcass_Bruising_and_Animal_Welfare_in_the_Transportation_of_Extensively_Reared_Beef_Cattle/links/5b852848299bf1d5a72cb381/Relationship-between-Methods-of-Loading-and-Unloading-Carcass-Bruising-and-Animal-Welfare-in-the-Transportation-of-Extensively-Reared-Beef-Cattle.pdf>
